# MRI evaluation of normal tissue deformation and breathing motion under an abdominal compression device

**DOI:** 10.1002/acm2.13165

**Published:** 2021-01-15

**Authors:** Maureen Lee, Anna Simeonov, Teo Stanescu, Laura A. Dawson, Kristy K. Brock, Michael Velec

**Affiliations:** ^1^ Department of Radiation Oncology Princess Margaret Cancer Centre University Health Network University of Toronto 610 University Avenue Toronto ON M5G 2M9 Canada; ^2^ TECHNA Institute University Health Network 100 College Street Toronto ON M5G 1L5 Canada; ^3^ Department of Imaging Physics The University of Texas MD Anderson Cancer Center Houston TX 77030 USA

**Keywords:** abdominal compression, breathing motion, organ deformation

## Abstract

**Purpose:**

Abdominal compression can minimize breathing motion in stereotactic radiotherapy, though it may impact the positioning of dose‐limiting normal tissues. This study quantified the reproducibility of abdominal normal tissues and respiratory motion with the use of an abdominal compression device using MR imaging.

**Methods:**

Twenty healthy volunteers had repeat MR over 3 days under an abdominal compression plate device. Normal tissues were delineated on daily axial T2‐weighted MR and compared on days 2 and 3 relative to day 1, after adjusting for baseline shifts relative to bony anatomy. Inter‐fraction organ deformation was computed using deformable registration of axial T2 images. Deformation > 5 mm was assumed to be clinically relevant. Inter‐fraction respiratory amplitude changes and intra‐fraction baseline drifts during imaging were quantified on daily orthogonal cine‐MR (70 s each), and changes > 3 mm were assumed to be relevant.

**Results:**

On axial MR, the mean inter‐fraction normal tissue deformation was > 5 mm for all organs (range 5.1–13.4 mm). Inter‐fraction compression device misplacements > 5 mm and changes in stomach volume > 50% occurred at a rate of 93% and 38%, respectively, in one or more directions and were associated with larger adjacent organ deformation, in particular for the duodenum. On cine‐MR, inter‐fraction amplitude changes > 3 mm on day 2 and 3 relative to day 1 occurred at a rate of < 12.5% (mean superior–inferior change was 1.6 mm). Intra‐fraction baseline drifts > 3 mm during any cine‐MR acquisition occurred at a rate of 23% (mean superior–inferior changes was 2.4 mm).

**Conclusions:**

Respiratory motion under abdominal compression is reproducible in most subjects within 3 mm. However, inter‐fraction deformations greater than 5 mm in normal tissues were common and larger than inter‐ and intra‐fraction respiratory changes. Deformations were driven mostly by variable stomach contents and device positioning. The magnitude of this motion may impact normal tissue dosimetry during stereotactic radiotherapy.

## 1 Introduction

Stereotactic body radiotherapy (SBRT) and MR‐based adaptive replanning are techniques being increasingly used to treat liver and pancreas tumors in an effort to further improve local control and toxicity outcomes.^1^, ^2^ Mitigating breathing motion in particular can permit smaller margins and dose escalation in the abdomen provided motion in dose‐limiting normal tissues are also accounted for.^3^, ^4^

Abdominal compression is commonly used to reduce breathing motion where breath‐hold or gating is unavailable or unsuitable. Using plate‐, corset‐, or belt‐based compression devices reduces the amplitude of abdominal targets including liver and pancreas tumors by 36–62% compared to free breathing measured using tumor surrogates on 4D CT, cone‐beam CT, or fluoroscopy.^3^, ^5^, ^6^, ^7^ On 4D cone‐beam CT, abdominal compression also improves inter‐ and intra‐fraction baseline liver reproducibility vs free breathing and most amplitude changes in liver amplitude are small (80% <3 mm).^8^, ^9^ Several studies have examined abdominal target motion directly using cine‐MR for improved temporal resolution and characterization of breathing patterns vs 4D CT‐based analyses.^10^, ^11^, ^12^, ^13^ Eccles et al. observed only a modest reduction in liver tumor amplitude on cine‐MR of 20% with compression compared to other imaging modalities with large inter‐patient variations in effectiveness of reducing amplitude.^14^

The inter‐fraction reproducibility of normal tissues is also important for their impact on SBRT planning. A clinical study of liver SBRT delivered with online MR guidance and gating under free breathing noted local failures possibly due to compromised target coverage adjacent to dose‐limiting normal organs.^2^ Online adaptive replanning for liver and pancreas SBRT may be required to enable isotoxic dose escalation to account for deformation within normal tissues and position within the abdomen.^15^, ^16^, ^17^ The impact of abdominal compression on normal tissue deformations is less clear in comparison to its impact on breathing motion. Only one study quantified liver deformation under abdominal compression with 9% liver volume on average deforming > 5 mm.^18^ This and other studies are limited by poor visibility of organ boundaries on cone‐beam CT and may not accurately quantify normal tissue motion.^19^

The purpose of this work is to assess the inter‐ and intra‐fraction reproducibility of upper abdominal normal tissues under an abdominal compression device, using repeat MR imaging which allows for improved organ at risk identification and delineation vs CT.^20^, ^21^ Characterizing inter‐fraction dose‐limiting normal tissue deformation and inter‐ and intra‐fraction respiratory motion will aid in understanding if resource‐intensive adaptive replanning strategies are warranted while using abdominal compression devices.

## 2 Methods

Twenty‐two healthy volunteers consented to this research ethics board‐approved imaging study. Data from 20 subjects who completed all imaging sessions were analyzed (60 axial MR, 120 cine‐MR images in total). Median age was 29 years (range 23–56) with an equal female–male ratio. Subjects were instructed to fast 2 hr prior to imaging. Three sessions were repeated over 3 days using a 1.5T MR simulator (Signa Twin Speed, GE, Milwaukee, WI) and eight‐channel torso coil. Subjects were positioned feet first supine, arms on chest, and immobilized with an abdominal compression plate device. Compression was performed using an in‐house‐developed MRI‐compatible device that has been documented elsewhere,^14^ which consists of a wooden, indexed frame with a compression plate that is secured by an adjustable screw to an arched support. Daily, compression plate was positioned midline and inferior to the xiphisternum. Pressure was determined by each subject’s maximum compression depth tolerated on day 1 and measured from the top of the plate to the bottom of the arch for reproducibility on subsequent days. Abdominal axial T2‐weighted single shot fast spin echo (SSFSE) sequences were first acquired under voluntary exhale breath‐hold with the following parameters (TE/TR = 90/1300, slice thickness = 5.0 mm, matrix 256 × 192, FOV ~ 360–420 mm) and a mean reconstructed resolution of 0.7 × 0.7 × 6.5 mm^3^.^14^ Sequential cine‐MR was then acquired in the mid‐liver sagittal and then coronal planes with the following parameters (echo time of 90 ms; repetition time of 1,300 ms) each approximately 70 s at 3 Hz (200–227 frames), and a mean reconstructed resolution of 1.4 × 1.4 × 10 mm^3^.^14^, ^22^

### 2.A Evaluation of organ reproducibility

T2‐weighted axial MR was analyzed in a treatment planning system (RayStation 6.1, RaySearch Laboratories, Stockholm, Sweden). The following regions of interest (ROI) were contoured using consensus guidelines for abdominal T2‐weighted MR^23^: liver, stomach, kidneys, duodenum (first plus second portions), pancreas, spleen, spinal canal. The external body and compression plate were also included as separate ROIs. A single observer contoured all normal tissues that were peer reviewed to minimize variability.

Images were initially rigidly registered about the vertebral column. Subsequently, automated rigid registration was done using soft‐tissue contours (liver, spleen, kidney ROIs) to account for and quantify baseline shifts in exhale position vs the vertebra prior to further analysis. Inter‐fraction motion was quantified for day 2 and 3 images relative to day 1 (baseline) using four metrics. First is the center of mass (COM) motion. Second and third are the mean surface distance to agreement (DTA) and Dice similarity coefficient (DSC) using the normal tissue contours to measure ROI surface displacement and volume overlap, respectively. Fourth is the mean deformation vector field (DVF) quantifying the residual volumetric deformation of each ROI. DVF was determined from deformable image registration performed between day 1 and subsequent images using a biomechanical model‐based algorithm and all anatomic ROI contours as input. This algorithm has been described previously and validated with subvoxel accuracy on abdominal MR.^24^ COM results were analyzed in the right/left (RL), anterior/posterior (AP), and superior/inferior (SI) directions or as the 3D vector magnitude for other metrics. Motion > 5 mm was assumed to be clinically relevant (i.e., exceeding average voxel dimension, registration accuracy, and contouring uncertainty). Variability in compression plate positioning and stomach volume changes was analyzed in detail for their potential impact on ROI deformation.

### 2.B Evaluation of respiratory reproducibility

**Fig. 1:**
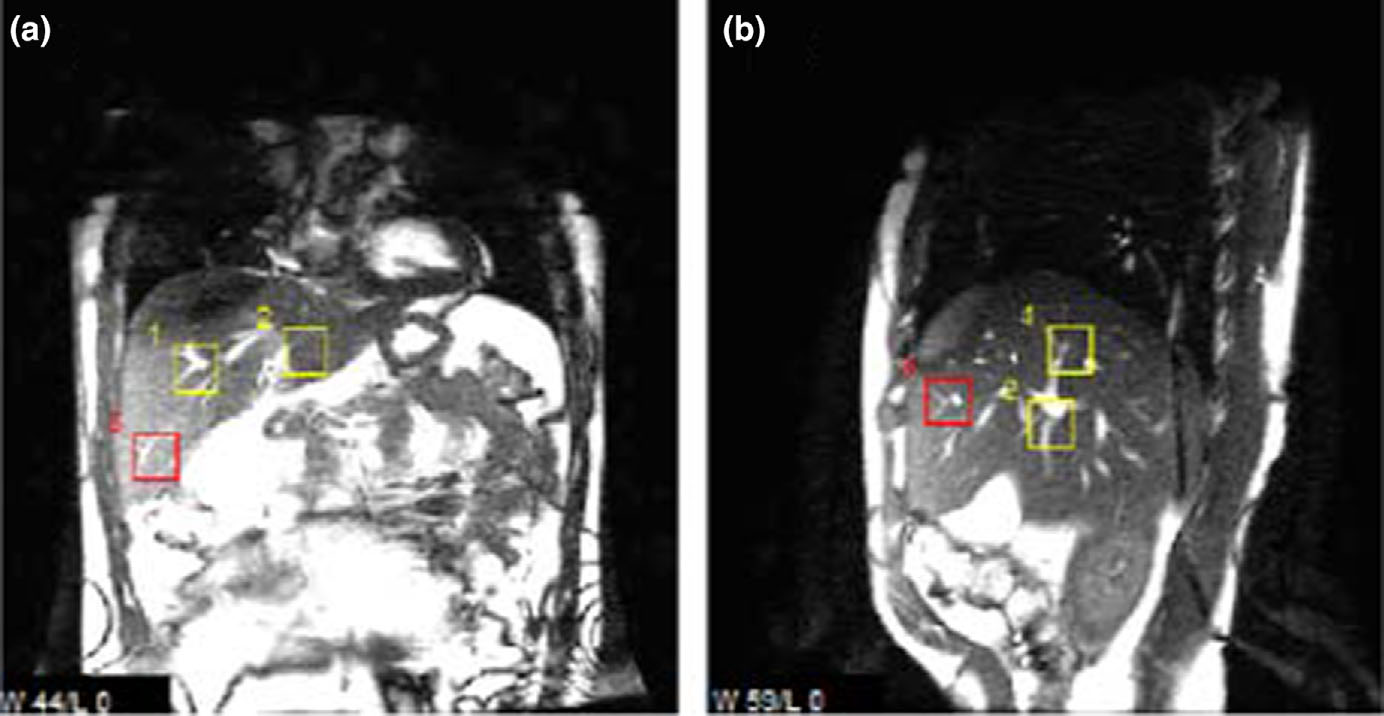
Cine‐MR in coronal (a) and sagittal (b) planes mid‐liver, showing user‐defined templates centered on prominent vessel bifurcations used for superior–inferior respiratory motion tracing.

Respiratory traces from cine‐MR were generated using a supervised automated algorithm tracking the frame‐to‐frame motion of three 16 × 16 mm, user‐defined templates centered on prominent vessel bifurcations visible in each case.^25^ Motion traces from the three templates were averaged for each imaging session to minimize out‐of‐plane motion and other artifacts (Fig. 1). Respiratory amplitude was quantified as the 5^th^–95^th^ percentile range of the SI motion averaged over the sagittal and coronal planes. Intra‐fraction baseline drifts were quantified as the range of the moving average of the prior 30 frames (~10 s) in each of the sagittal and coronal planes. Inter‐fraction changes in amplitude > 3 mm between day 2 and 3 relative to day 1 and intra‐fraction baseline drifts > 3 mm observed on any of the daily motion traces were assumed to be clinically relevant as this impacts typical planning margins.^14^

## 3 Results

Following rigid registration of the vertebra, the overall mean ± SD (range) absolute COM displacements of the compression plate ROI from days 2 and 3 relative to day 1 were 12.4 ± 9.5 (1.0–36.1) mm RL, 7.0 ± 6.5 (0–30.7) mm AP, and 11.0 ± 8.1 (1–33.4) mm SI. The frequency of displacements > 5 mm was 80% RL, 48% AP, and 75% SI over all image sets (or 93% in one or more directions), occurring in all 20 volunteers. The plate was most reproducible in the AP direction of applied compression.

The overall mean ± SD (range) absolute COM motion accounting for baseline shifts (i.e., combined soft‐tissue ROIs vs vertebra) was 1.2 ± 1.1 (0–5.5) mm RL, 1.6 ± 1.5 (0.1–7.6) mm AP, and 4.5 ± 4.7 (0–22.9) mm SI. Baseline shifts > 5 mm occurred at a rate of 3% RL, 3% AP, and 35% SI over all images, occurring in 5 of 20 volunteers.

Volunteer geometric characteristics are in the Supplementary Table.

### 3.A Inter‐fraction normal tissue reproducibility

The 3D residual motion and deformation for all ROIs under abdominal compression after adjusting for baseline shifts are shown in Table 1. Based on the ROI contour analysis, the mean distance to agreement (DTA) was > 5 mm on average for the stomach only, while the average 3D COM motion was ≥ 5 mm for all normal tissues. From the deformable image registrations, the mean 3D deformation was > 5 mm on average for the stomach, duodenum, pancreas, and spleen (range 8.7–13.4 mm). Greater than 50% of the normal tissues deformed by > 5 mm on average (range 53–100%), except the spinal canal and kidneys. Overall, the stomach, duodenum, and pancreas had the largest deformations and most often exceeding 5 mm in magnitude.

**Table 1:** Inter‐fraction motion and deformation of normal tissues described over all patients and all axial T2 images.

Normal tissue	3D mean DTA		3D mean COM		3D mean DVF			DSC
	mean ± SD (mm)	Frequency > 5 mm (%)	mean ± SD (mm)	Frequency > 5 mm (%)	mean ± SD (mm)	Frequency > 5 mm (%)	Mean volume deforming > 5 mm (%)	mean ± SD
Liver	3.1 ± 1.2	8	4.7 ± 2.6	38	5.8 ± 2.3	53	49 ± 29	0.91 ± 0.04
Stomach	7.6 ± 3.9	65	11.9 ± 6.4	90	13.4 ± 6.4	100	87 ± 14	0.63 ± 0.16
Duodenum	4.5 ± 2.6	28	9.9 ± 6.2	73	9.6 ± 5.8	75	74 ± 31	0.52 ± 0.19
Pancreas	4.4 ± 2.3	25	9.2 ± 5.8	83	8.7 ± 4.7	83	69 ± 29	0.56 ± 0.16
Right Kidney	2.6 ± 1.3	8	5.0 ± 3.5	28	5.1 ± 2.9	35	41 ± 36	0.86 ± 0.06
Left Kidney	2.7 ± 1.6	10	5.2 ± 4.1	33	5.6 ± 3.7	40	43 ± 39	0.84 ± 0.10
Spleen	3.7 ± 2.7	23	8.4 ± 7.8	60	9.0 ± 7.5	63	62 ± 39	0.78 ± 0.16
Spinal canal	1.6 ± 0.9	0	7.0 ± 4.9	55	5.7 ± 4.5	40	39 ± 44	0.79 ± 0.10

Abbreviations: COM = center of mass; DSC = Dice similarity coefficient; DTA = distance to agreement; DVF = deformation vector field; SD = standard deviation.

The average volume change for all organs across the three scans for males and females was found to be similar at 17% and 14%, respectively. No trend was observed between separation magnitude and volume change magnitude. Absolute mean (range) changes in stomach volume were 73% or 181cc (1–580% or 3–730cc) and changes > 50% occurred at a rate of 38%, occurring in 12 of 20 subjects.

**Fig. 2:**
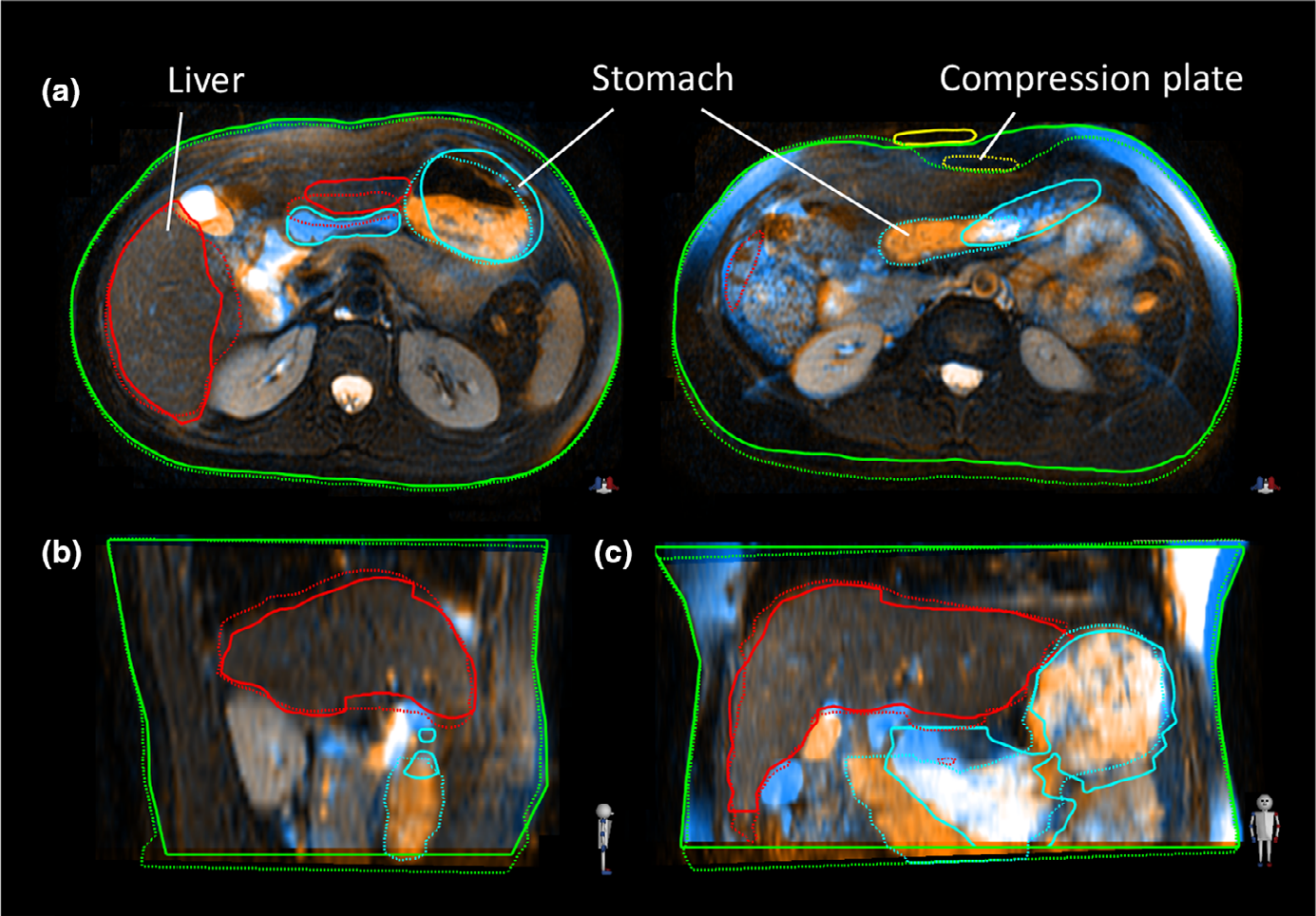
Example of inter‐fraction variations between day 1 (solid contours) and day 3 (dashed contours): (a) axial images at the level of the mid‐liver (left) and superior compression plate right; (b) sagittal and coronal (c) images mid‐liver.

**Fig. 3:**
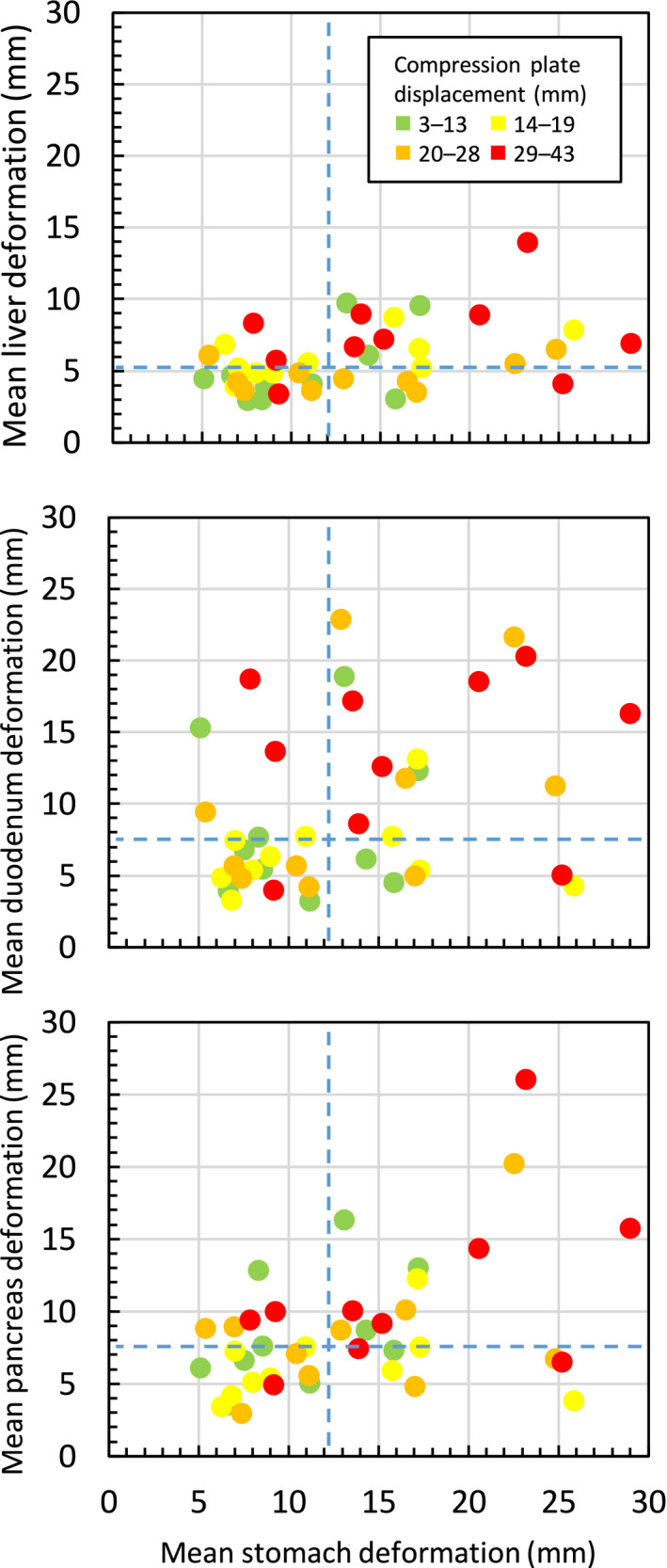
Relationship between inter‐fraction stomach deformation and plate displacements on deformation of other organs. Each point is the mean inter‐fraction organ deformation for a participant on repeat MR on days 2 and 3 (40 images total). Dashed blue lines indicate median deformation magnitude over all participants.

Larger stomach deformations (mean 18.6 vs 8.2 mm) were significantly associated with larger mean deformations of the liver, duodenum, and pancreas (range 2–5 mm, *P* ≤ 0.005). There was a trend for larger absolute 3D COM displacements of the compression plate (mean 28.3 vs 12.6 mm) to be associated with larger mean deformation of the duodenum (4.4 mm, *P* = 0.02) and pancreas (3.5 mm, *P* = 0.07). Plate displacements and stomach deformations were not correlated (R^2^ = 0.08) and both were associated with deformation in other organs (Figs. 2, 3). Figure 3 illustrates that smaller compression plate displacements (3–13 mm) resulted in reduced liver, duodenum, and pancreas deformations (<5 mm) in 70%, 40%, and 20% of cases, respectively. Table 2 shows the risk of substantial ROI deformations with large compression plate motion or stomach deformation.

**Table 2:** Risk (%) of inter‐fraction mean deformation > 5 mm on repeat imaging (40 images) when the compression plate is misplaced by > 20 mm and/or the stomach has mean deformation > 12 mm.

Normal tissue	No plate misplacement or stomach deformation (N = 12)	Either plate misplacement or stomach deformation (N = 16)	Both plate misplacement and stomach deformation (N = 12)
Liver	25%	63%	67%
Duodenum	67%	69%	92%
Pancreas	75%	81%	92%
Right Kidney	25%	31%	50%
Left Kidney	17%	31%	75%
Spleen	42%	69%	75%
Spinal canal	42%	44%	33%

### 3.B Respiratory reproducibility

Mean ± SD (range) breathing amplitude averaged over three imaging sessions was 2.0 ± 1.0 (0–3.0) mm RL, 3.3 ± 1.4 (0–6.6) mm AP, and 8.4 ± 2.6 (0–4.7) mm SI. Mean absolute inter‐fraction changes in amplitude were ≤ 1.6 mm in each directions, with maximum changes of 3.0 mm LR, 6.6 mm AP, and 4.7 mm SI. Amplitude changes > 3 mm occurred at a rate of 2.5% RL, 5% AP, and 7.5% SI over all image sets, occurring in 4 of 20 subjects.

**Fig. 4:**
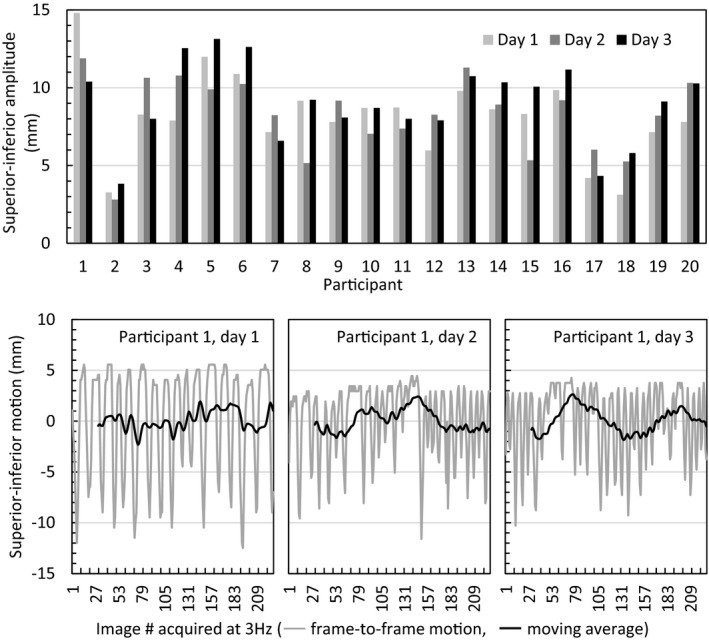
Inter‐fraction superior–inferior breathing amplitude on cine MR (5^th^–95^th^ percentile) for all participants (top). Example of cine MR motion over ~ 75 s in participant #1 with intra‐fraction baseline drifts (moving average) >3 mm each day (bottom).

Mean absolute intra‐fraction baseline drifts were ≤ 2.4 mm in each direction, with maximum drifts of 2.8 mm LR, 2.3 mm AP, and 4.7 mm SI. Drifts > 3 mm occurred only in the SI direction at a rate of 23%, occurring in 8 of 20 subjects. Amplitude variations and baseline drifts are shown in Fig. 4.

## 4 Discussion

This is the first study conducted to the authors’ knowledge quantifying inter‐fraction deformation of multiple normal tissues under an abdominal compression plate device using MR. Additional strengths of this work include the large participant sample size, repeated multi‐plane cine‐MR and 3D T2‐weighted axial MR and application of a validated deformable registration model. The use of abdominal compression resulted in reproducible day‐to‐day breathing amplitude and baseline drifts < 3 mm in most subjects. Substantial day‐to‐day deformations exceeding the prespecified 5 mm threshold were observed in most normal tissues on average due in part to device positioning and variable stomach contents despite the use of fasting instructions. As radiotherapy practice shifts toward the dose escalation or SBRT in the treatment of abdominal cancers, accounting for variations in dose‐limiting normal tissues will become increasingly important.

The stomach, duodenum, and pancreas had the largest deformations with mean DVF values of 9–13 mm on average. Most other organs also had significant volumes deforming by > 5 mm. Variations in compression plate positioning occurred in nearly all subjects and correlated to some degree of normal tissue deformation (Fig. 3). The plate position was most reproducible in the direction of compression (AP) and misplacements along the body surface (RL being the largest and most frequent) may have resulted from an inability to index the plate to the body surface (e.g., from a lack of tattoos). Misplacements of this magnitude may indicate a need for implementation of clearer indexing protocols. Figure 2 illustrates how a surface misplacement of the plate results in internal tissue deformation despite being compressed to the same magnitude. Large compression plate displacements and consequential organ deformations observed in our study may justify incorporation of new institutional workflows to measure plate displacements during daily treatment imaging.

The use of abdominal compression has been widely used in radiotherapy for cancers of the lung, liver, pancreas.^3^, ^14^, ^26^, ^27^ Our institution has been reluctant to use compression for pancreatic radiotherapy due to the perception it increases contact with the healthy duodenum. The current study demonstrates substantial day‐to‐day deformation under compression. Mampuya *et al*. showed compression actually increases inter‐fraction variations in tumor position.^26^ Benzodiazepines can alternatively reduce breathing motion alone or in combination with abdominal compression,^6^, ^28^ potentially eliminating stronger applied pressures and minimizing inter‐fraction setup variations.

Inter‐fraction breathing amplitude was largely consistent on repeat cine‐MR imaging in this study. However, intra‐fraction baseline drifts in mean liver position of > 3 mm were seen in 23% of cine‐MR acquisitions. The ~ 70 s duration of the cine‐MR imaging in this study may not be representative of a longer abdominal treatment. Cusumano *et al* observed drifts up to 16 mm for MR‐guided treatments lasting upward of 30 min.^10^ Real‐time tumor tracking or “tumor trailing” to account for time‐averaged drifts in tumor position is in development for MR‐guided radiotherapy and can potentially mitigate in their dosimetric impact.^29^ Gating using electromagnetic transponders can permit similar gains without MR; however, they are often implanted at some distance from tumors.^30^

The participation of healthy volunteers lacking comorbidities seen in patients is a potential limitation of this study as these may impact setup reproducibility. Dietary preparation guidelines may not have been rigidly adhered to resulting in mean stomach volume changes of 33% (range −77 to 580%). Other studies have shown fasting instructions to be largely ineffective at minimizing inter‐fraction stomach changes in patients treated with radiotherapy.^31^ El‐Bared *et al* reported mean stomach changes of 53% (−47 to 334%) in pancreas patients treated with online MR‐guided adaptive SBRT following a 3‐hour fasting window.^15^ Study results also rely on accurate contouring aided by MR, consensus guidelines, and review by multiple observers. Although the MR slice thickness was slightly larger than currently used in the clinic (~5 mm) substantial deformation was often observed exceeding the 6.5 mm slices in all organs to varying degrees.

Quantifying the need for replanning when using abdominal compression was not feasible in the healthy volunteers and it is possible the normal tissue deformation observed may not have a dosimetric impact in true patients, depending on the target location and technique used. However, the study by El‐Bared *et al* reported adaptation was beneficial in 50% of cases where 3‐mm planning margins were applied and patients were treated in free‐breathing.^15^ Our prior study accumulating delivered doses on cone‐beam CT during liver SBRT reported significant dose deviations in normal tissue dose vs planning under abdominal compression.^19^ The deviations relative to planning ranged from −38% to 10% in maximum dose for luminal gastrointestinal organs, −14% to 13% for minimum tumor dose, and −2 to 9% for liver NTCP. Approximately half of these dose deviations were a result of inter‐fraction organ deformation or changes in breathing amplitude. Normal tissue deformations may have been underestimated, however, due to the poor visibility of nonliver tissues on cone‐beam CT. Comparing the equivalent population geometry metrics from our prior cone‐beam CT analysis,^19^ the current MR analysis shows increased normal tissue deformation of 22–51% in magnitude in the liver, stomach, and kidneys, and 189–219% for the stomach and duodenum. In the current study, the duodenum (considered dose‐limiting) exhibited differential motion > 3 mm in one or more directions in 95% of cases vs the pancreas and 90% vs the liver. Taken together, this suggests adaptive replanning to spare normal tissues or target dose escalation is likely still required when abdominal compression is used in the majority of cases. Unpredictable peristaltic motion in luminal gastrointestinal organs has also been recently reported ranging from 3 to 10 mm, similar in magnitude to respiratory motion.^13^ For longer online MR‐guided adaptation, such motion may require real‐time monitoring which is also beneficial for baseline drifts. In the majority of centers without online replanning capabilities, the motion results from this study can inform to potentially design planning risk volumes for non‐adaptive radiotherapy.

## 5 Conclusions

Substantial day‐to‐day deformations > 5 mm were observed in all normal tissues on MR under an abdominal compression plate in healthy volunteers, and are larger than previous estimates based on cone‐beam CT. Deformations were associated to abdominal compression device mispositioning and stomach content variations despite fasting instructions. The magnitude of normal tissue deformations observed in our study may impact SBRT dosimetry in patients with upper abdominal cancer.

## Conflicts of Interest

LA Dawson and KK Brock have a licensing agreement with RaySearch Laboratories.

## Author Contribution

ML, AS, LAD, KKB, and MV substantially contributed to the conception or design of the work; AS, LAD, KKB, and MV contributed to the acquisition of data; ML, TS, LAD, KKB, and MV contributed to data analysis; ML, TS, LAD, KKB, and MV involved in the interpretation of data for the work. ML, AS, TS, LAD, KKB, and MV drafted the work or revised it critically for important intellectual content; ML, AS, TS, LAD, KKB, and MV gave the final approval of the version to be published. Agreement to be accountable for all aspects of the work in ensuring that questions related to the accuracy or integrity of any part of the work are appropriately investigated and resolved by ML, AS, TS, LAD, KKB, and MV.
