# Supplementary material for: MRI evaluation of normal tissue deformation and breathing motion under an abdominal compression device
Source: J Appl Clin Med Phys. 2021 Jan 15;22(2):90–7. doi: 10.1002/acm2.13165 (PMC7882116; doi:10.1002/acm2.13165)
Supplement: Supplementary file 1 [file ACM2-22-90-s001.pdf]

Supplemental Table. Subject separation in AP and RL directions at the level of the centre of the compression plate, and organ volumes on day 1 MR images.

M/F	lbs	Separation		Volume (cc)						
		AP (cm)	RL (cm)	Stomach	Duodenum	Liver	Pancreas	Spleen	Kidney	Canal
M	160	14.1	24.8	590.9	48.9	1408.7	82.2	197.0	154.0	29.9
	152	12.9	27.1	133.3	25.9	1326.9	45.4	120.1	131.0	22.6
	150	16.2	25.0	241.8	29.3	1522.6	59.8	176.7	128.0	29.4
	211	19.8	25.3	960.9	38.8	1998.5	70.5	245.8	195.0	35.8
	230	20.6	29.2	280.0	30.7	2115.5	87.7	271.9	138.0	32.2
	162	15.5	24.3	168.7	22.6	1328.4	70.1	159.1	162.0	32.2
	187	16.8	25.7	568.9	18.7	1623.9	76.8	280.7	218.0	37.5
	200	22.7	24.1	276.6	28.0	1703.7	56.1	256.3	105.0	30.1
	165	23.4	25.1	600.6	21.4	1269.3	39.2	161.2	44.0	19.0
	150	12.9	21.0	187.7	21.8	1399.3	79.8	258.7	155.0	29.1
F	186	20.1	24.9	188.6	9.5	1438.3	43.4	202.9	88.0	25.4
	108	12.1	18.7	249.4	8.2	979.8	52.9	121.3	106.0	27.7
	150	15.2	25.1	347.4	16.4	1389.8	54.7	314.9	179.0	39.6
	120	14.7	20.7	256.3	18.5	1127.1	42.1	122.7	127.0	29.3
	135	14.9	22.8	562.7	47.5	1409.2	51.3	203.5	113.0	33.5
	115	12.3	18.2	283.0	21.9	1021.9	31.3	138.5	125.0	33.7
	145	14.7	22.7	287.7	21.6	1053.1	51.2	215.2	108.0	28.0
	155	15.7	24.6	403.6	17.5	1458.8	42.2	22.5	118.0	35.2
	127	17.3	17.9	480.1	34.8	1018.8	75.4	168.0	120.0	34.1
	140	15.7	17.8	133.4	23.4	1077.5	83.8	117.4	152.0	35.1
